# Adhesins of *Leptospira interrogans* Mediate the Interaction to Fibrinogen and Inhibit Fibrin Clot Formation *In Vitro*

**DOI:** 10.1371/journal.pntd.0002396

**Published:** 2013-08-29

**Authors:** Rosane Oliveira, Renan F. Domingos, Gabriela H. Siqueira, Luis G. Fernandes, Natalie M. Souza, Monica L. Vieira, Zenaide M. de Morais, Silvio A. Vasconcellos, Ana L. T. O. Nascimento

**Affiliations:** 1 Centro de Biotecnologia, Instituto Butantan, São Paulo, São Paulo, Brazil; 2 Programa Interunidades em Biotecnologia, Instituto de Ciências Biomédicas, Universidade de São Paulo, São Paulo, Brazil; 3 Laboratorio de Zoonoses Bacterianas do VPS, Faculdade de Medicina Veterinária e Zootecnia, Universidade de São Paulo, São Paulo, Brazil; University of California San Diego School of Medicine, United States of America

## Abstract

We report in this work that *Leptospira* strains, virulent *L. interrogans* serovar Copenhageni, attenuated *L. interrogans* serovar Copenhageni and saprophytic *L. biflexa* serovar Patoc are capable of binding fibrinogen (Fg). The interaction of leptospires with Fg inhibits thrombin- induced fibrin clot formation that may affect the haemostatic equilibrium. Additionally, we show that plasminogen (PLG)/plasmin (PLA) generation on the surface of *Leptospira* causes degradation of human Fg. The data suggest that PLA-coated leptospires were capable to employ their proteolytic activity to decrease one substrate of the coagulation cascade. We also present six leptospiral adhesins and PLG- interacting proteins, rLIC12238, Lsa33, Lsa30, OmpL1, rLIC11360 and rLIC11975, as novel Fg-binding proteins. The recombinant proteins interact with Fg in a dose-dependent and saturable fashion when increasing protein concentration was set to react to a fix human Fg concentration. The calculated dissociation equilibrium constants (K*_D_*) of these reactions ranged from 733.3±276.8 to 128±89.9 nM for rLIC12238 and Lsa33, respectively. The interaction of recombinant proteins with human Fg resulted in inhibition of fibrin clot by thrombin-catalyzed reaction, suggesting that these versatile proteins could mediate Fg interaction in *Leptospira*. Our data reveal for the first time the inhibition of fibrin clot by *Leptospira* spp. and presents adhesins that could mediate these interactions. Decreasing fibrin clot would cause an imbalance of the coagulation cascade that may facilitate bleeding and help bacteria dissemination

## Author Summary

Leptospirosis is probably the most widespread zoonosis in the world. Caused by spirochaetes of the genus *Leptospira*, it has greater incidence in tropical and subtropical regions. The disease has become prevalent in cities with sanitation problems and a large population of urban rodent reservoirs, which contaminate the environment through their urine. Understanding the mechanisms involved in pathogenesis of leptospirosis should contribute to new strategies that would help fight the disease. We show in this work that *Leptospira* strains, virulent, attenuated or saprophytic are capable of binding fibrinogen (Fg). The interaction of leptospires with Fg inhibits the formation of fibrin clot that may result of an imbalance in the haemostatic equilibrium. In addition, we show that plasminogen (PLG)/plasmin (PLA) generation on the surface of leptospires can lead to Fg degradation, showing evidence of possible route of fibrinolysis in leptospirosis. We also present six leptospiral proteins, as novel Fg-binding proteins, capable of inhibiting fibrin clot formation by thrombin-catalyzed reaction, suggesting that in *Leptospira* these multifunctional proteins could mediate Fg interaction. Our data suggest possible mechanisms that leptospires could employ to affect the coagulation cascade and fibrinolytic system that might lead to bacteria spreading.

## Introduction

The spirochete *Leptospira interrogans* is a highly invasive pathogen and the causal agent of leptospirosis, one of the most widespread zoonosis of human and veterinary concern [1], [2]. The transmission occurs through contact with environmental water contaminated by leptospires shed in the urine of animal carriers [1], [3]. Humans are accidental and terminal hosts in the transmission process of leptospirosis. The leptospires enter the body via abrasions on skin or actively through mucosa, spreading to any tissue, and colonizing target organs [4], [3]. *Leptospira* can cause damage of the endothelium of small blood vessels, leading to hemorrhage and localized ischemia in multiple organs. As a consequence, renal tubular necrosis, hepatocellular damage and development of leptospirosis-associated pulmonary hemorrhage syndrome (LPHS) may occur in the host [1], [5], [6]. The mechanisms responsible for bleeding in leptospirosis are poorly understood. Hemolysins could play an important role in this toxic response and several genes coding for predicted hemolysins were identified in the genome sequencing *L. interrogans* [7]. Yet, when evaluated, these proteins failed to show hemolytic activity in human erythrocytes [8], [9].

Fg and PLG are key proteins in the coagulation cascade and fibrinolysis, respectively, and critical determinants of bacterial virulence and host defense [10]. Fg is the major clotting protein present in blood plasma with an important role in blood coagulation and thrombosis. PLG under the action of its activators generates PLA, a serine-protease capable of degrading ECM components, fibrin, facilitating the pathogen penetration and invasion [11].

We have previously shown that PLA-associated *Leptospira* renders the bacteria with proteolytic activity capable of degrading ECM components [12] that in turn, may help bacterial penetration and dissemination. Furthermore, PLA-coated leptospires have also shown to degrade IgG and C3b that could facilitate the bacterial immune evasion [13]. The adhesion of physiological osmotically induced *Leptospira* with Fg was described but their effect on fibrin formation was not ascertained [14]. The leptospiral proteins LigB and OmpL37 were shown to interact with Fg and LigB was reported to reduce fibrin clot formation [15], [16], [17].

We thus decided to evaluate if Fg-associated *Leptospira* was capable to inhibit the fibrin clot formation and the ability of seven recombinant proteins to act as leptospiral Fg-receptors. We report that *Leptospira* strains and the recombinant ECM- and PLG-interacting proteins, rLIC12238 [18], Lsa33, Lsa25 [19], Lsa30 [20], OmpL1 [21], rLIC11360 and rLIC11975 [22], are capable to adhere to Fg. We also show that this interaction inhibits fibrin clot formation by thrombin-catalyzed reaction. Moreover, PLA-coated *Leptospira* was capable to degrade Fg. Altogether, the results suggest possible pathways that *Leptospira* may interfere with the coagulation/bleeding process.

## Methods

### Ethics statement

All animal studies were approved by the Ethical Committee for Animal Research of Instituto Butantan, São Paulo, SP, Brazil under protocol n^o^ 798/11. The Committee in Animal Research in Instituto Butantan adopts the guidelines of the Brazilian College of Animal Experimentation.

### *Leptospira* strains and culture conditions

The non-pathogenic *L. biflexa* serovar Patoc strain Patoc 1, the pathogenic attenuated *L. interrogans* serovar Copenhageni strain M-20 and the virulent strains of *L. interrogans* serovar Kennewicki strain Pomona Fromm (LPF) and serovar Copenhageni strain Fiocruz L1-130 were cultured at 28°C under aerobic conditions in liquid EMJH medium (Difco) with 10% rabbit serum, enriched with L-asparagine (wt/vol: 0.015%), sodium pyruvate (wt/vol: 0.001%), calcium chloride (wt/vol: 0.001%), magnesium chloride (wt/vol: 0.001%), peptone (wt/vol:0.03%) and meat extract (wt/vol: 0.02%) [23]. *Leptospira* cultures are maintained in Faculdade de Medicina Veterinária e Zootecnia, USP, São Paulo, SP, Brazil. Unless otherwise stated, experiments were performed with leptospires resuspended in low salt – lsPBS (50 mM), instead of PBS that contains 137 mM NaCl, because it is an osmolarity condition closer to cultivation, which is approximately 35 mM.

### Immunofluorescence assay

For the immunofluorescence assay (IFA), live *L. interrogans* sorovar Copenhageni strain M-20 suspensions (∼10^8^) were harvested at 5,000× g for 15 min, washed three times with lsPBS, resuspended in 200 µL lsPBS, and fixed with 200 µL with 4% paraformaldehyde for 40 min at 30°C. After the fixation, propidium iodide (Sigma) diluted 1∶50 in lsPBS was added to stain the nuclei, and the suspensions were incubated for 45 min at 30°C. After this time, the leptospires were gently washed three times with lsPBS and blocked with lsPBS containing 5% BSA for 90 min at 30°C, followed by incubation with 16 µg Fg in 200 µL lsPBS for 60 min at 37°C. The leptospires were washed three times with lsPBS plus 1% BSA and incubated with goat-produced antiserum against human Fg at a 1∶50 dilution for 45 min at 37°C. Leptospires were washed three times again and incubated with rabbit anti-goat IgG antibodies conjugated to fluorescein isothiocyanate (FITC; Sigma) at a dilution of 1∶400 for 45 min at 37°C. After this incubation, the leptospires were washed twice with lsPBS containing 1% BSA and once with distilled water. Finally they were resuspended in lsPBS-antifading solution (ProLong Gold; Molecular Probes). The immunofluorescence-labeled leptospires were examined by use of a confocal LSM 510 META immunofluorescence microscope (Zeiss, Germany). As a control for unspecific binding, Fg was absent from the reaction mixtures.

### Binding of soluble human Fg to *Leptospira*

For accessing the leptospiral binding to soluble human Fg, ELISA plates were coated with 10^8^ leptospires in lsPBS per well (*L. interrogans* sorovar Copenhageni strain Fiocruz L1-130, *L. interrogans* serovar Copenhageni strain M-20, and *L. biflexa* serovar Patoc strain Patoc 1), allowed to set for 3 h at 37°C and then blocked with 200 µL of phosphate-buffered saline containing 0.05% Tween (PBS-T) with 10% non-fat dry milk. After 2 h incubation, plates were washed with PBS-T and then 100 µL of a Fg solution (Fg- Sigma) (10 µg/mL in lsPBS) were added per well and incubation proceeded for 2 h. After extensive washing, 100 µL of solution containing goat anti-Fg (1∶25,000 in lsPBS) were added per well and incubation was carried out for 1 h at 37°C. After three washings, 100 µL of solution containing peroxidase (HRP)-conjugated rabbit anti-goat IgG (1∶50,000 in lsPBS) were added per well and the reaction continued for 1 h at 37°C. The wells were washed three times, and *o*-phenylenediamine (OPD) (1 mg/mL) in citrate phosphate buffer (pH 5.0) plus 1 µL/mL H_2_O_2_ was added (100 µL per well). The reaction was allowed to proceed for 10 min and interrupted by the addition of 50 µL of 8 M H_2_SO_4_. Readings were taken at 492 nm in a microplate reader (Multiskan EX; Thermo Fisher Scientific, Helsinki, Finland). The binding of Fg to each leptospiral strain was performed in triplicate and a negative control in which Fg was omitted was included in the experiment. In the no-cell control, BSA replaced leptospires. For statistical analyses, the binding of Fg to different strains and control BSA was performed using one-way ANOVA, followed by Tukey post-test for pairwise comparisons.

### Effect of Fg-bound leptospires on fibrin clot formation

Live leptospires (*L. interrogans* sorovar Copenhageni strain Fiocruz L1-130, *L. interrogans* serovar Copenhageni strain M-20, and *L. biflexa* serovar Patoc strain Patoc 1), 10^9^, 10^7^, 10^5^ cells/mL were harvested at 5,000× g for 20 min at room temperature, washed once with lsPBS, resuspended in 0.5 mL of lsPBS plus 1 mg/mL of Fg (Sigma), and incubated for 2 h at 37°C. ELISA plates were placed with 90 µL/well of leptospires plus Fg and 10 µL/well of thrombin (10 U/mL - Sigma). The fibrin clot formation was measured every 1 min for 10 min and then every 5 min for 35 min by an ELISA plate reader (Multiskan EX Thermo Fisher Scientific) at OD595_nm_. The positive control of the reaction employed Fg (1 mg/mL) plus thrombin (10 U/mL) while in the negative control thrombin was omitted. For statistical analyses, inhibition of fibrin clot formation was performed by one-way ANOVA, followed by Tukey post-test for pairwise comparisons. Three independent experiments were performed.

### Human Fg degradation by leptospires coated with PLA

Virulent *L. interrogans* serovar Kennewicki strain Pomona Fromm (10^8^ leptospires/sample) were treated in 200 µL lsPBS with the addition of: (a) 10 µg PLG (b) 3U urokinase (uPA) (c) 10 µg PLG and 3U uPA (generating PLA-coated leptospires) or (d) only lsPBS with no additions (untreated). The bacteria were incubated for 1 h at 37°C with the PLG, and for one more hour after the addition of uPA. The cells were washed three times with lsPBS to remove the free PLG, uPA and PLA. Then, the bacteria were resuspended in 100 µL lsPBS containing goat anti-human Fg human purified Fg (Sigma, USA) as substrate, and incubated for 16 h at 37°C. Three additional controls were employed: (a) one sample received 1 µg of the protease inhibitor aprotinin (Sigma, USA) to the Fg-leptospires incubation, (b) one sample contained only Fg without leptospires, and (c) one sample received the PLG and uPA treatment without the addition of leptospires to rule out the free PLA Fg degradation. The leptospires were removed by centrifugation, 20 µL of the supernatants were separated by 8% SDS-PAGE and then transferred to nitrocellulose membranes in semi-dry equipment. The membranes were blocked by incubating overnight at 4°C with 5% non-fat dry milk and 1% BSA. The Fg detection was performed by incubations with goat anti-human Fg antibodies and rabbit anti-goat secondary antibodies conjugated with HRP, followed by ECL (GE Healthcare) development.

In another assay, after the incubation for Fg degradation, the pelleted leptospires and the remaining cell-free supernatants were also evaluated for the presence of Fg by ELISA. The cells were resuspended in 100 µL lsPBS and coated onto ELISA plates overnight at 4°C, as well as the cell-free supernatants previously diluted 20 times. The plates were washed three times with PBS-T and blocked with solution containing 5% BSA and 5% non-fat dry milk for 2 h at 37°C, following incubations with anti-Fg polyclonal antibodies (1∶25,000) and secondary antibodies conjugated to peroxidase (1∶50,000). The reactions were developed by addition of 100 µL/well of solution containing 1 mg/mL OPD and 1 µL/mL H_2_O_2_, and stopped by addition of 50 µL/well H_2_SO_4_. The samples in which the leptospires received no treatment (only PBS) were considered as having 100% of Fg for comparative purposes.

### Bioinformatics characterization of the proteins

Predicted coding sequences (CDSs) were analyzed for their cellular localization by PSORT program, http://psort.nibb.ac.jp [24], [25]. The web servers SMART, http://smart.embl-heidelberg.de/ [26], [27], PFAM, http://www.sanger.ac.uk/Software/Pfam/ [28], and LipoP, http://www.cbs.dtu.dk/services/LipoP/ [29] were used to search for predicted functional and structural domains within the amino acid sequences of the CDSs.

### Cloning, expression and purification of recombinant proteins

Amplification of the CDSs was performed by PCR from *L. interrogans* serovar Copenhageni strain M-20 genomic DNA using complementary primer pairs (Table 1). The gene sequences were amplified without the signal peptide tag and cloned into pAE expression vector [30]. The final constructs were verified by DNA sequencing on an ABI Prism 3730_L sequencer (Seq- Wright, Houston, TX) with appropriate vector-specific T7 (F: TAATACGACTCACTATAGGG) and pAE (R:CAGCAGCCAACTCAGTTCCT) primers. Detailed of cloning, expression and purification of the recombinant proteins Lsa33, Lsa25, rLIC12238, Lsa30, OmpL1, rLIC11360 and rLIC11975 has been previously described [18], [19], [21], [20], [22]. The proteins rLIC11360 and Lsa30 were kept at pH 12 required for their solubility.

**Table 1:** Gene locus, protein name, gene bank reference sequence, protein conservation, sequence of the primers employed for DNA amplification, and molecular mass of expressed recombinant proteins.

Gene locus^1^	Given name	NCBI reference sequence number^2^	Description/ function	Conservation (identity)^3^	Sequence of primers for PCR amplification	Recombinant protein molecular mass
						
						
LIC11834	Lsa33^a^	YP_001783.1	Putative lipoprotein	Lai (99%)	F:5′CTCGAGGATCTACAAGGTGGGGTTT	33.10 kDa
				LBH (87%)	TTAC3′(XhoI)	
				LSS (88%)	R:5′CCATGGTTACTGAGGTTTTACTTGG	
				LlicsVM (60%)	TCC3′ (NcoI)	
				LBP (31%)		
LIC11975	Lsa36^b^	YP_0019141	Outer membrane protein	Lai (99%)	F: 5′ CTCGAGGAATGTAGCGGTGCGG3′	36.12 kDa
				LBH (0%)	(XhoI)	
				LSS (0%)	R: 5′AAGCTTTCATCCCAAAAGATAA T	
				LlicsVM (54%)	TCA3′ (HindIII)	
				LBP (38%)		
LIC10973	OmpL1^c^	YP_000947.1	Outer membrane Protein	Lai (94%)	F:5′GGATCCAAAACATATGCAATTGTA	32.40 kDa
				LBH (87%)	GG3′ (BamHI)	
				LSS (87%)	R: 5′GGTACCTTAGAGTTCGTGTTTATA	
				LlicsVM (51%)	ACC3′ (KpnI)	
				LBP (44%)		
LIC11360	Lsa23^b^	YP_0013241	Putative lipoprotein	Lai (99%)	F: 5′GGATCCGAACTTCCTTACTTTTCC	22.53 kDa
				LBH (69%)	CCTAAC3′ (BamHI)	
				LSS (67%)	R: 5′AAGCTTGAATGTTGACTAGAGGC	
				LlicsVM (44%)	ATTTACT3′ (HindIII)	
				LBP (0%)		
LIC12238	rLIC12238^d^	YP_002173	Hypothetical protein	Lai (99%)	F: 5′CTCGAGTGTTTTAAACCTACCGG	17.63 kDa
				LBH (77%)	AG3′ (XhoI)	
				LSS (0%)	R: 5′AAGCTTCTACTTCATCGCTTTTTC	
				LlicsVM (50%)	TATATC 3′ (HindIII)	
				LBP (39%)		
LIC11087	Lsa30^e^	YP_0010571	Hypothetical protein	Lai (99%)	F: 5′CTCGAGGGAGATTCCAGAAAGAA	30.00 kDa
				LBH (75%)	AAC3′ (XhoI)	
				LSS (81%)	R: 5′AAGCTTACCTGTGTGATGCGCTT C	
				LlicsVM (29%)	T3′(HindIII)	
				LBP (30%)		
LIC12253	Lsa25^a^	YP_002188.1	Hypothetical protein	Lai (100%)	F:5′CTCGAGGAGGAGAAACCGGACGA	24.07 kDa
				LBH (77%)	TAC 3′ (XhoI)	
				LSS (79%)	R:5′CCATGGTTAGGGAAGACTTCTAAC	
				LlicsVM (33%)	ACATC 3′ (NcoI)	
				LBP (39%)		

1 http://aeg.lbi.ic.unicamp.br/world/lic/;

2 http://www.ncbi.nlm.nih.gov/protein/;

3 http://www.ncbi.nlm.nih.gov/blast/Blast.cgi/. This work, Lai: *L. interrogans* serovar Lai [56]; LBH: *L. borgpetersenii* serovar Hardjo-bovis [57]; LBP: *L. biflexa* serovar Patoc [58]; LlicsVM: *L. licerasiae* serovar Varillal [59]; LSS: *L. santarosai* serovar Shermani [60].

a Previously published by Domingos *et al.*, 2012 [19].

b Previously published by Siqueira *et al.*, 2013 [22].

c Previously published by Fernandes *et al.*, 2012 [21].

d Previously published by Vieira *et al.*, 2010 and Oliveira *et al.*, 2011 [18], [36].

e Previously published by Souza *et al.*, 2012 [20].

### Antiserum

Five female BALB/c mice (4–6 weeks old) were immunized subcutaneously with 10 µg of each recombinant protein adsorbed in 10% (vol/vol) of Alhydrogel (2% Al(OH)_3_, Brenntag Biosector, Denmark), used as adjuvant. Two subsequent booster injections were given at 2-week intervals with the same recombinant proteins preparation. Negative - control mice were injected with PBS plus Alhydrogel. Two weeks after each immunization, the mice were bled from the retro - orbital plexus and the pooled sera were analyzed by ELISA for determination of antibody titers.

### Fg binding assay and dose-response curves

The binding of the recombinant proteins to Fg was evaluated by a modified ELISA, as follows: 96-well plates (Costar High Binding, Corning) were coated overnight in PBS at 4°C with 100 µL of 10 µg/mL of the human Fg (Sigma); LigB7-12 protein of *L. interrogans*, known as having Fg-binding domain [31], [17], generous donation from Dr. Odir Dellagostin, UFPEL, RS, Brazil, was employed as a positive control. PspA, an outer membrane protein of *Streptococcus pneumoniae* [32], kind gift from Dr. Luciana Leite, Instituto Butantan, SP, Brazil, was employed as an unrelated negative control protein. Gelatin (Difco) was also employed as negative protein control. Plates were washed three times with PBS-T and blocked for 2 h at 37°C with PBS with 10% (wt/vol) non-fat dry milk. The blocking solution was discarded and 100 µL of 10 µg/mL recombinant proteins in PBS was incubated for 2 h at 37°C. Wells were washed three times with PBS-T and incubated for 1 h at 37°C with polyclonal mouse antiserum produced against each recombinant protein. The serum dilution used was determined by titration curve with the corresponding recombinant protein and the value of 1.0 at OD492_nm_ was employed. These values are: 1∶1,000 for rLIC12238; 1∶750 for Lsa33; 1∶500 for rLIC11975 and rLIC11360; 1∶400 for Lsa30, 1∶800 for OmpL1, 1∶500 for Lsa25 and 1∶200 for PspA. After incubation, plates were washed again and incubated with HRP-conjugated anti-mouse immunoglobulin G (IgG), diluted 1∶5,000 in PBS. After three washings, 100 µL/well of 1 mg/mL OPD plus 1 µL/mL H_2_O_2_ in citrate phosphate buffer (pH 5.0) were added. The reactions were carried out for 15 min and stopped by the addition of 50 µL/well of H_2_SO_4_ (8 M). Readings were taken at OD492 _nm_. In another assay, the assessment of bound proteins was performed by incubation for 1 h at 37°C with monoclonal anti-polyhistidine-HRP (Sigma) at appropriate dilutions: 1∶5,000 for rLIC12238; 1∶10,000 for rLIC11975 and rLIC11360; 1∶1,000 for Lsa30; 1∶500 for Lsa33, 1∶400 for OmpL1, 1∶200 for Lsa25 and 1∶200 for LigB7-12. The reaction was developed with 1 mg/mL OPD plus 1 µL/mL H_2_O_2_, as described above. The rate of interaction of recombinant proteins to Fg was determined by measuring the reaction as a function of time. The OD492_nm_ value after 2 h interaction was considered the maximal binding (100%) and was used for statistical analyses, using Student's two-tailed t test. For determination of dose-response curves of the binding of recombinant proteins to human Fg, protein concentrations varying from 0 to 4,000 nM in PBS were used. Binding was detected with polyclonal antibodies against each protein at the dilution described above, except for LigB7-12 where monoclonal anti-polyhistidine-HRP was used (1∶200). For statistical analyses, the binding of recombinant proteins to human Fg was compared to its binding to gelatin by Student's two-tailed t test.

### Antibody inhibition assay

96-well plates were coated with 1 µg of Fg in 100 µL of PBS and allowed to set overnight at 4°C. The wells were washed three times with PBS-T and then blocked with 200 µL of 10% (wt/vol) nonfat dry milk for 2 h at 37°C. Prior to the next step, the proteins were incubated for 1 h at 37°C with the respective antibodies diluted 1∶50 in 100 µL of PBS. After the incubation, each recombinant protein (1 µg) was added per well in 100 µL of PBS, and allowed to attach to Fg for 90 min at 37°C. Polyclonal serum obtained in mice against another leptospiral protein, DnaK (1∶50), was employed as a control that is not specific for the studied proteins. After washing six times with PBS-T, bound recombinant proteins were detected by adding monoclonal HRP-conjugated mouse anti-polyhistidine-HRP (Sigma) at dilutions described above. Incubation proceeded for 1 h at 37°C. The detection was performed with OPD, as previously described. BSA or gelatin was used as negative control (data not shown). For statistical analyses, the attachment of blocked recombinant proteins to Fg was compared to the binding with the untreated proteins by the two-tailed t test (* *P*<0.05).

### Effect of recombinant protein denaturing on the interaction with Fg

ELISA plates were coated overnight at 4°C with 100 µL of 10 µg/mL Fg. Plates were washed three times with PBS-T and blocked with 200 µL of 10% (wt/vol) nonfat dry milk for 2 h at 37°C. The recombinant proteins were denatured by incubation at 96°C for 10 min; 1 µg of each was added per well in 100 µL of PBS. The recombinant proteins were allowed to attach to Fg at 37°C for 90 min. After washing six times with PBS-T, bound recombinant proteins were detected by incubation with mouse serum raised against the respective protein (dilutions described above) at 37°C for 1 h. After three washings with PBS-T, 100 µL of a 1∶5,000 dilution of HRP-conjugated rabbit anti-mouse IgG (Sigma) in PBS was added per well for 1 h at 37°C. The detection was performed with OPD, as previously described. BSA or gelatin was used as negative control (data not shown). For statistical analyses, the attachment of denatured recombinant proteins to Fg was compared to untreated recombinants binding by the two-tailed t test (* *P*<0.05).

### Dissociation equilibrium constant (K*_D_*) for the binding of recombinant proteins to human Fg

The ELISA data were used to calculate the dissociation equilibrium constant (K*_D_*) according to the method previously described [33] based on the equation: K*_D_* = (Amax.[protein])/A) – [protein], where A is the absorbance at a given protein concentration, Amax is the maximum absorbance for the ELISA plate reader (when the equilibrium is reached), [protein] is the protein concentration and K*_D_* is the dissociation equilibrium constant for a given absorbance at a given protein concentration (ELISA data point).

### Fibrin clot formation inhibition assay in the presence of recombinant proteins

The assay of thrombin-catalyzed fibrin clot inhibition was performed in the presence of recombinant proteins. We have employed the concentration of recombinant proteins in which there was a binding saturation to Fg. Proteins that do not bind Fg were employed at the same concentration range. Protein concentration employed: Lsa33 – 3,000 nM, OmpL1 – 3,000 nM, rLIC12238 – 3,500 nM, rLIC11975 – 2,000 nM, LigB7-12 – 1,500 nM, Lsa25 – 3,000 nM, PspA – 3,000 nM. Recombinant proteins were resuspended in 0.5 mL of PBS plus 1 mg/mL of Fg and incubated for 2 h at 37°C. ELISA plates were coated with 90 µL/well of recombinant proteins plus Fg and 10 µL/well of thrombin (10 U/mL). The fibrin clot formation was measured as previously described. Reduction of the fibrin clot formation was calculated by comparing the value of the last reading point, at 45 min, with the positive control (100%). Analysis was performed using one-way ANOVA, followed by Tukey post-test for pairwise comparisons.

### Inhibition of live leptospires binding to Fg by recombinant proteins

ELISA plates were coated with Fg (1 µg/well) in PBS and allowed to set overnight at 4°C; the wells were then washed and blocked with 10% non-fat dry milk in PBS-T for 2 h at 37°C. The blocking solution was discarded, and the wells were incubated for 90 min at 37°C with increasing concentrations of recombinant proteins (0 to 4.5 µM). After three washings, 100 µL/well of 4×10^7^ live *L. interrogans* serovar Copenhageni strain M-20 were added for 90 min at 37°C. The unbound leptospires were washed and the quantification of bound leptospires was performed indirectly by anti-LipL32 antibodies produced in mice (1∶4,000), based on the fact that LipL32 is a major expressed membrane leptospiral protein [34]; the procedure was followed by the addition of HRP-conjugated anti-mouse IgG antibodies, essentially as described in Atzingen et al., (2008) [35]. The detection was performed by OPD, as above described.

## Results

### Binding of human Fg by *L. interrogans* cells

**Figure 1:**
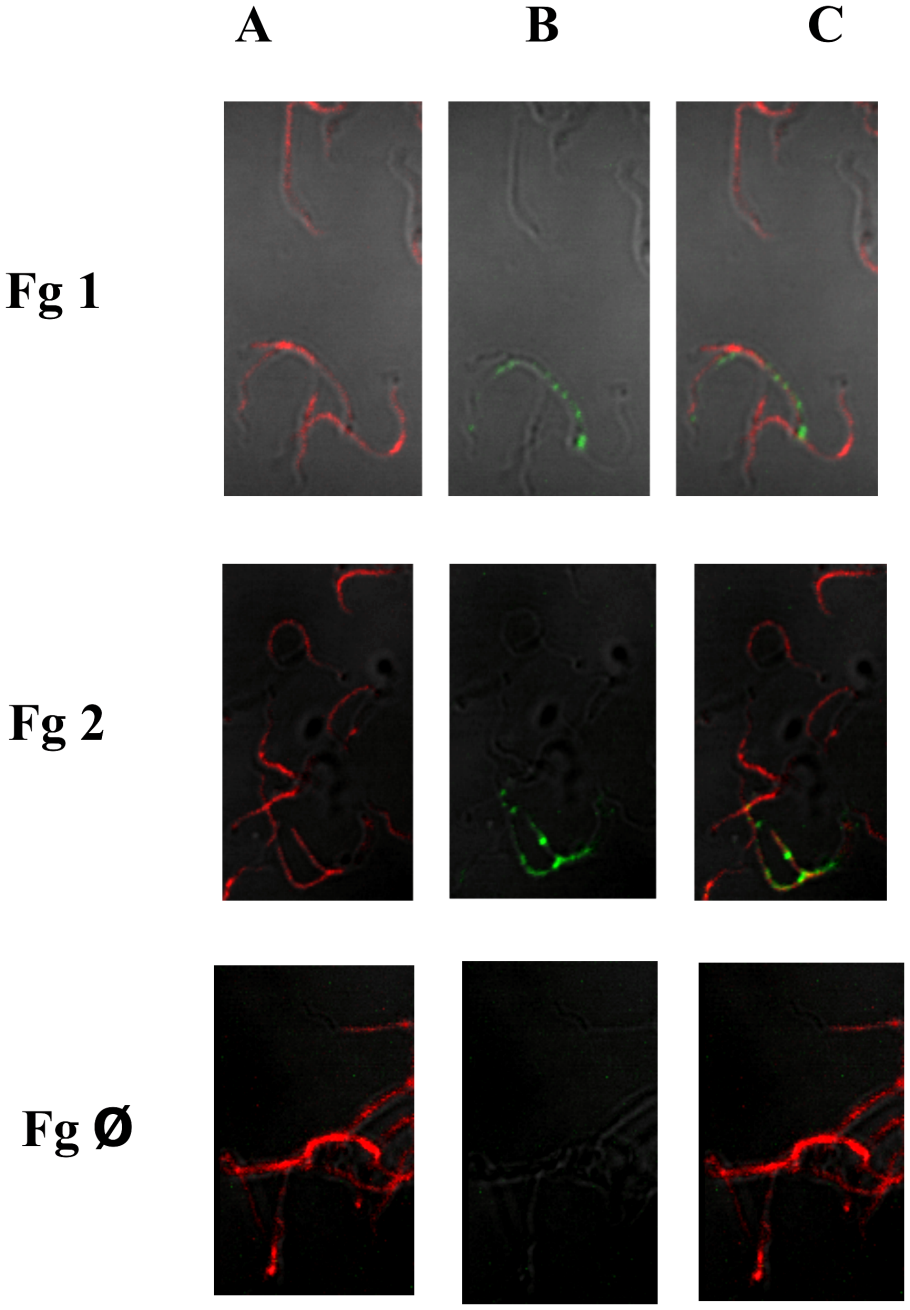
Recognition of Fg binding to *Leptopsira* by IFA. Live *L. interrogans* sorovar Copenhageni strain M-20 were fixed with 2% paraformaldehyde and treated wit 16 µg of Fg. The recognition was assessed through polyclonal anti-Fg antibodies under confocal immunofluorescence microscope (Fg1 and Fg2). Control in which Fg was absent from the incubation mixture is shown (Fg Ø). Panels A: DNA propidium iodide-stained, B: FITC-stained and C: A+B composite images.

The ability of *L. interrogans* sorovar Copenhageni strain M-20 cells to bind human Fg was performed by immunofluorescence assay (IFA). Leptospires were visualized by propidium iodide staining (Fig. 1, panel A) followed by protein detection with goat anti- human Fg, in the presence of anti-goat IgG antibodies conjugated to FITC. Green fluorescence could be observed for Fg (Fig. 1 -Fg1B, Fg2B). The localization of the protein-green light within the leptospires was achieved by superimposing both fields and the results obtained are shown in Fig. 1 - Fg1C and Fg2C. The FgØ shows the control of the reaction in which Fg was absent.

### Interaction of human Fg with *Leptospira* spp. and inhibition of fibrin clot formation

**Figure 2:**
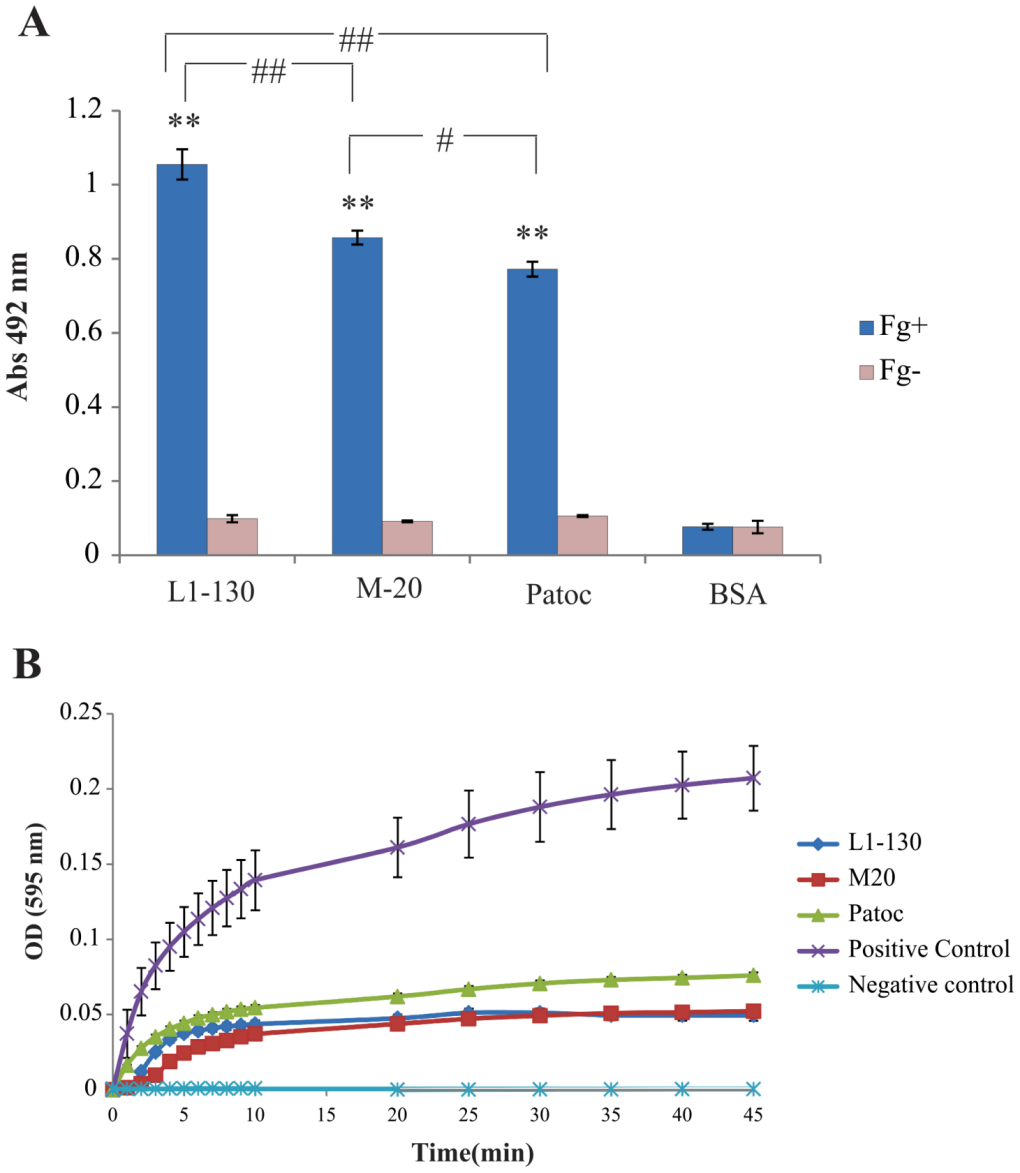
(A). Interaction of leptospires to Fg. The binding of soluble human Fg (10 µg/mL) to immobilized strains of leptospires (*L. interrogans* serovar Copenhageni strain Fiocruz L1-130, *L. interrogans* serovar Copenhageni strain M-20, and *L. biflexa* serovar Patoc strain Patoc 1) was accessed by ELISA. After 2 h interaction, plates were washed and the detection of bound Fg was verified by incubation with goat anti-Fg (1∶25,000) followed by incubation with HRP-conjugated rabbit anti-goat IgG (1∶50,000). The binding of Fg to each leptospiral strain was performed in triplicate and the data represent the mean ± the standard deviation from one representative experiment. Fg+ and Fg− stand for the presence and absence of Fg, respectively, while in the no-cell control, BSA replaced leptospires. For statistical analyses, the attachment of Fg to leptospiral strains was performed by one-way ANOVA followed by Tukey post-test for pairwise comparison, asterisks above the bars refer to comparison to BSA (no cell control) (**P<0.01). The comparison among leptospiral strains is also shown (#P<0.05 and ## P<0.01). (**B**) Inhibition of fibrin clot formation by leptospires. 1 mg/mL of Fg plus live leptospires, pre-incubated for 2 h at 37°C, and 10 U/mL of thrombin were placed into ELISA plates. The positive control of the reaction employed Fg (1 mg/mL) plus thrombin (10 U/mL) while in the negative control thrombin was omitted. BSA was used as a negative control of binding. The fibrin clot formation was measured every 1 min for 10 min and then every 5 min for 35 min. Each point was performed in triplicate and expressed as the mean absorbance value at 595_nm_ ± SD for each point.

We have evaluated the capability of soluble human Fg (10 µg/mL) to interact with immobilized strains of leptospires by ELISA. We performed the experiments using virulent *L. interrogans* serovar Copenhageni strain Fiocruz L1-130, pathogenic attenuated *L. interrogans* serovar Copenhageni strain M-20 and one saprophytic *L. biflexa* serovar Patoc strain Patoc 1. The binding of Fg to each leptospiral strain was performed in triplicate and the data represent the mean ± the standard deviation from one representative experiment is depicted in Fig. 2A. The measurements were performed in the presence (Fg+) and absence (Fg−) of human Fg while in the no-cell control, BSA replaced leptospires. For statistical analyses, the attachment of Fg to leptospiral strains was performed by one-way ANOVA followed by Tukey post-test for pairwise comparison, asterisks above the bars refer to comparison to BSA (no cell control) (**P<0.01). The comparison among leptospiral strains is also shown (#P<0.05 and ## P<0.01). The results show that all strains tested were able of binding human Fg, but the virulent strain was more efficient. We have assessed the effect of *Leptospira*-bound to Fg on the inhibition of thrombin-catalyzed fibrin clot formation. The reaction was analyzed with the same strains and readings were taken at OD595_nm_ every 1 min for the first 10 min and then every 5 min for 35 min. The complete reaction, Fg plus thrombin, was used as a positive control while in the negative one, thrombin was missing. The determination was performed in two independent experiments and a representative assay is shown in Fig. 2B. The data show that all the strains studied promoted an inhibition of fibrin formation statistically significant compared to the reaction positive control. However, the small difference observed between the strains pathogenic and saprophyte was not statistically relevant.

### PLA-associated leptospires on human Fg

**Figure 3:**
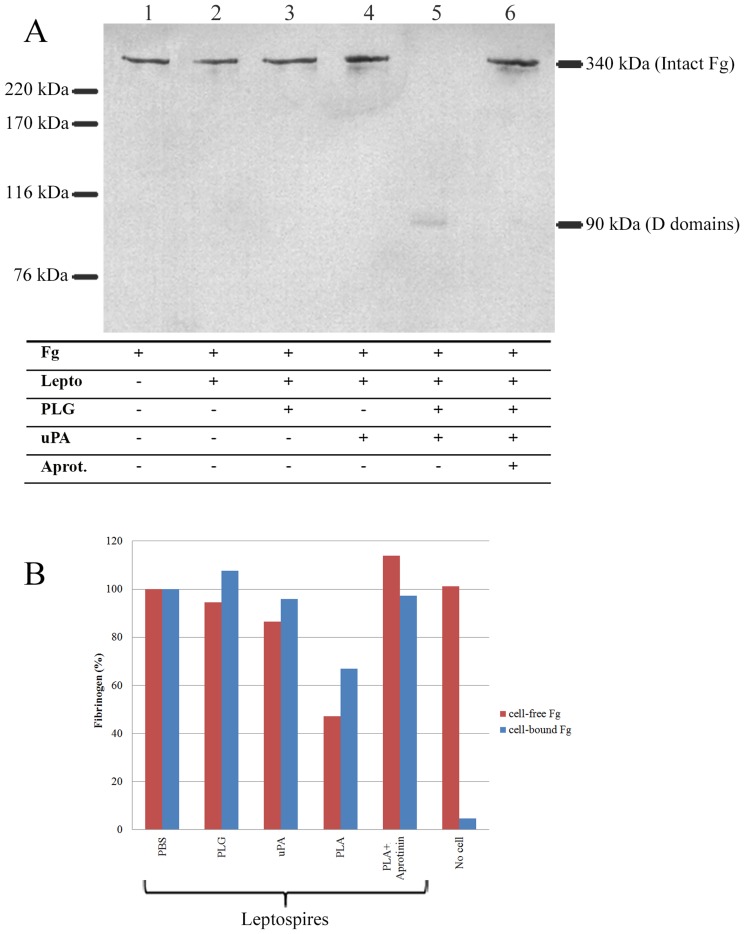
Degradation of Fg by PLA-coated leptospires. (**A**) *L. interrogans* serovar Kennewicki strain Pomona Fromm (LPF) (10^8^/sample) were treated with the addition of 10 µg PLG (lane 3), 3U uPA (lane 4) 10 µg PLG and 3U uPA (PLA) (lanes 5, 6) or no additions (untreated) (lane 2). Control lacking leptospires (lane1) and addition of 1 µg aprotinin to the Fg-leptospires incubation (lane 6) were employed. The cells were incubated with 15 µg/mL human purified Fg for 16 h at 37°C, and the supernatants were fractioned by SDS-PAGE and transferred to membranes. The Fg was detected by specific antibodies and the reaction was developed by ECL reagent. Indicated on the left are the molecular mass standards, and on the right, the masses of the intact Fg (340 kDa) and its degradation product (D domains, 90 kDa). (**B**) After treatments and incubation with Fg, the cell-free (supernatants) and cell-bound Fg were coated onto ELISA plates and detected by Fg polyclonal antibodies. A control lacking leptospires received the PLG+uPA treatment to rule out the contribution of contaminating cell-free PLA to Fg degradation.

The effect of PLA-coated virulent *L. interrogans* serovar Kennewicki strain Pomona Fromm on human Fg was evaluated by Western blotting using anti-human Fg antibodies (Fig. 3A). The results show that PLA generation on the surface of *Leptospira* cause degradation of human Fg (Fig. 3A, lane 5), which is not observed when at least one of the reaction components is missing (Fig. 3A, lanes 1, 2, 3and 4) and completely prevented in the presence of a serine protease inhibitor, aprotinin (Fig. 3A, lane 6). The data suggest that PLA-coated leptospires were capable to employ their proteolytic activity to interfere with one of the coagulation cascade substrate.

The Fg degradation by PLA-coated leptospires was also evaluated by ELISA. When leptospires were treated with PLG+uPA, the detection of Fg remaining in cell-free solution is decreased when compared to untreated, only PLG or only uPA-treated or when aprotinin was added to the Fg incubation (Fig. 3B). An additional control lacking leptospires was added in order to rule out the contribution of free PLA to the Fg degradation. As expected, the data showed that the remaining Fg level in this control was comparable with the non-proteolytic controls. Cell-bound Fg was also evaluated. PLA-coated leptospires retain Fg binding ability, though it seems to be diminished (Fig. 3B), probably due to Fg degradation.

### Selection of putative surface proteins from genome sequences

The rationale for protein selection was mostly based on cellular localization, since surface proteins are potential receptors for Fg. We have selected seven proteins, all of them previously shown to be leptospiral adhesins and were already published, rLIC12238 [18], [36], Lsa33, Lsa25 [19], Lsa30 [20], OmpL1 [21], rLIC11360 and rLIC11975 [22]. Table 1 summarizes features of the selected proteins, gene locus, given name, gene conservation within the sequenced genomes, the sequences of primers used for cloning techniques and molecular mass.

### Expression and purification of recombinant proteins

The amplified coding sequences, excluding the signal peptide tags, were cloned and expressed as full-length proteins in *E. coli*. The recombinant proteins were expressed with 6×His tag at the N-terminus and purified by nickel affinity chromatography, as previously described [35].

### Interaction of leptospiral recombinant proteins with Fg

**Figure 4:**
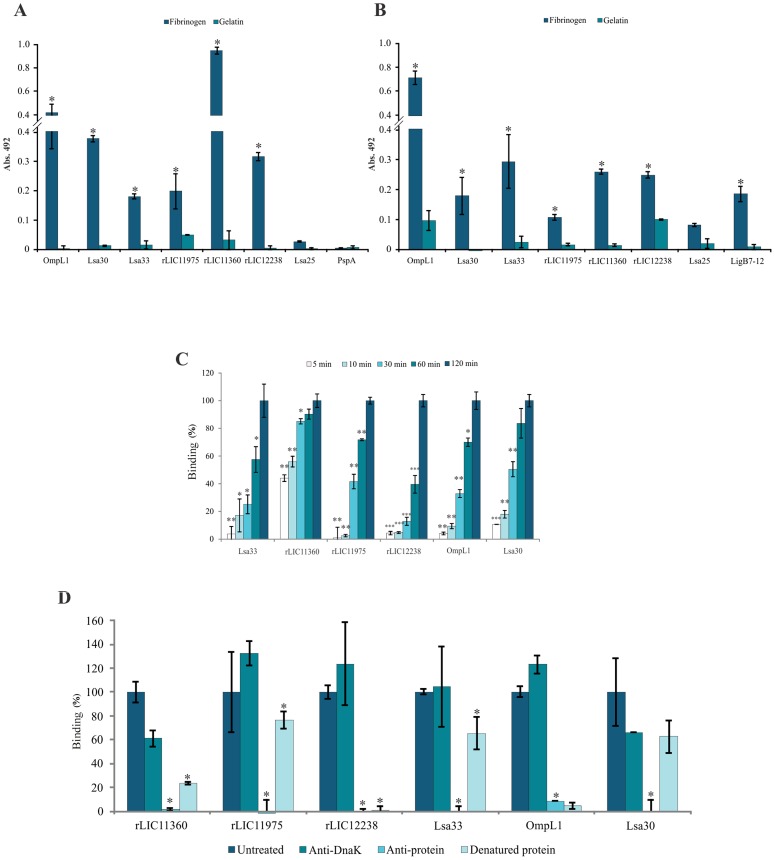
Recombinant proteins binding to human Fg. Human Fg (10 µg/mL) was coated to ELISA plates and allowed to interact with the recombinant proteins (10 µg/mL). Gelatin was used as a negative control for nonspecific binding. The reaction was detected with polyclonal antibodies raised against each protein, including anti-DnaK, used as non-specific antibodies (M&M), in (**A**), (**C**) and (**D**) while monoclonal anti-polyhistidine HRP antibodies was employed in (**B**). In (**C**) the percentage of binding for each protein with Fg was measured as a function of time up to 2 h. The statistical analysis was performed by two-tailed *t* test (*P<0.05) compared to the binding after 2 h. The effect of denaturing the recombinant proteins by incubation for 10 min at 96°C or blocking the proteins with the respective antibodies for 1 h at 37°C, previously to interaction with Fg is shown in (**D**). Bars represent the mean of absorbance at 492_nm_ ± the standard deviation of three replicates for each protein and are representative of three independent experiments. For statistical analyses, the binding of recombinant proteins to human Fg was compared to its binding to gelatin or untreated recombinant binding, in the case of (**D**) by two-tailed *t* test (*P<0.05).

Proteins of *Leptospira* have been reported to bind Fg [15], [16], [17]. We thus decided to investigate whether the selected surface-exposed proteins were capable of binding human Fg *in vitro*. Seven recombinant leptospiral proteins, expressed and purified in our laboratory, LigB7-12, used as positive control [31], [16], [17], and the negative protein controls gelatin and pneumococcal recombinant protein PspA, were individually placed onto 96-wells and incubated with previously immobilized human Fg. The binding was quantified by ELISA and the results obtained from three independent experiments are shown in Fig. 4. The protein bound to Fg was probed with the respective homolog polyclonal antiserum raised in mice (Fig. 4A) or with the monoclonal antibody anti-histidine tag (Fig. 4B). The percentage of binding for each protein with Fg, measured as a function of time is shown in Fig. 4C. The protein rLIC11360 promptly reacted with Fg with 40% of binding achieved after 5 min reaction, contrasting with rLIC11975 that showed very low binding activity at this time (Fig. 4C). The binding of recombinant proteins to Fg was also assessed after blocking the proteins with the corresponding antibody and after submitting them to denaturing conditions at 96°C for 10 min (Fig. 4D). The binding was totally inhibited by antibody-blocked protein in the case Lsa30, Lsa33, rLIC11975, rLIC12238 and rLIC11360, while a very low percentage of the binding remained for OmpL1 (8.4%), suggesting the participation of non- immunogenic epitopes on the interaction (Fig. 4D). Anti-DnaK serum, control employed as unrelated antibody, promoted a partial decrease on the binding of rLIC11360 and Lsa30, no effect with the protein Lsa33, while a slightly increase was detected with the proteins rLIC11975, rLIC12238 and OmpL1. At any rate, these differences were not statistically significant and are probably due to the presence of non-specific antibodies in the polyclonal serum. The adhesion of heat-denatured proteins to Fg was almost totally abolished in the case of rLIC12238 and OmpL1, 24% remained with rLIC11360, while 63–77% of the binding continued with Lsa30, Lsa33 and rLIC11975 (Fig. 4D). The results suggest that for some proteins the binding depends on their conformational structures while with others it probably relies on their linear primary conformation.

### Characterization of the binding of recombinant proteins to Fg

**Figure 5:**
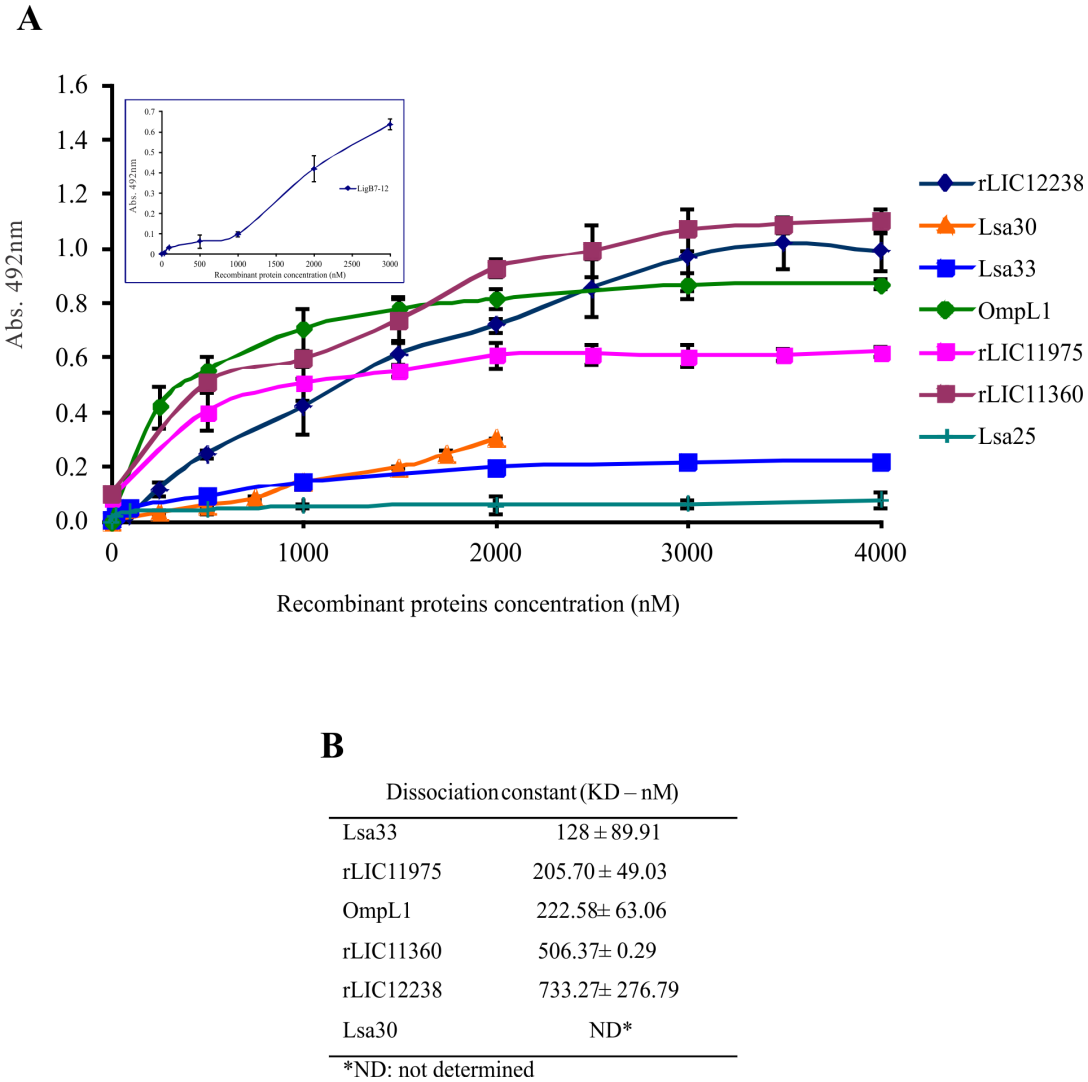
Binding characterization of recombinant proteins to human Fg. (**A**) Ten µg/mL of Fg was immobilized into 96-wells ELISA plates and 0 to 4,000 nM of each recombinant protein was added for interaction. The bindings were detected using antiserum raised in mice against each protein followed by HRP-conjugated anti-mouse IgG. Gelatin was employed as negative control for each assayed protein (not shown) and Lsa25, as a leptospiral recombinant protein negative control. Data represent the mean absorbance values ± the standard deviation of three replicates for each experimental group of three independent experiments. The insert shows the dose response curve obtained with 10 µg/mL immobilized Fg and 0 to 3,000 nM LigB7-12, employed as leptospiral Fg-binding protein. The binding was detected with monoclonal anti-polyhistidine HRP antibodies. (**B**) The dissociation constant (K*_D_*) was calculated based on ELISA data for the recombinant proteins that have reached the equilibrium concentrationMean absorbance values at 492_nm_ (± the standard deviations of three independent experiments) were compared to those obtained with untreated Fg (0 mM).

The interactions between the recombinant proteins and Fg was assessed on a quantitative basis, as indicated in Fig. 5A. Dose-dependent and saturable binding was observed when increasing concentrations (0 to 4,000 nM) of recombinant proteins rLIC12238, Lsa33, OmpL1, rLIC11975 and rLIC11360, or (0 to 2,000) of Lsa30, were allowed to individually adhere to a fixed human Fg amount (10 µg/mL) for 2 h. Saturation was reached with all except Lsa30 protein, due to the impossibility to achieve this protein at higher concentrations. In the case of LigB7-12, dose-response curve was measured using monoclonal anti-His tag antibodies, and the results depicted in the insert of Fig. 5A shows that saturation was not reached in the protein concentration range employed. Based on the ELISA data, the calculated dissociation equilibrium constants (K*_D_*) for the recombinant proteins with Fg are depicted in Fig. 5B; the highest and the lowest K*_D_* values were for rLIC12238 (733.3±276.8 nM) and Lsa33 (128±89.9 nM), respectively.

### Inhibition of fibrin formation by recombinant proteins-bound to Fg

**Figure 6:**
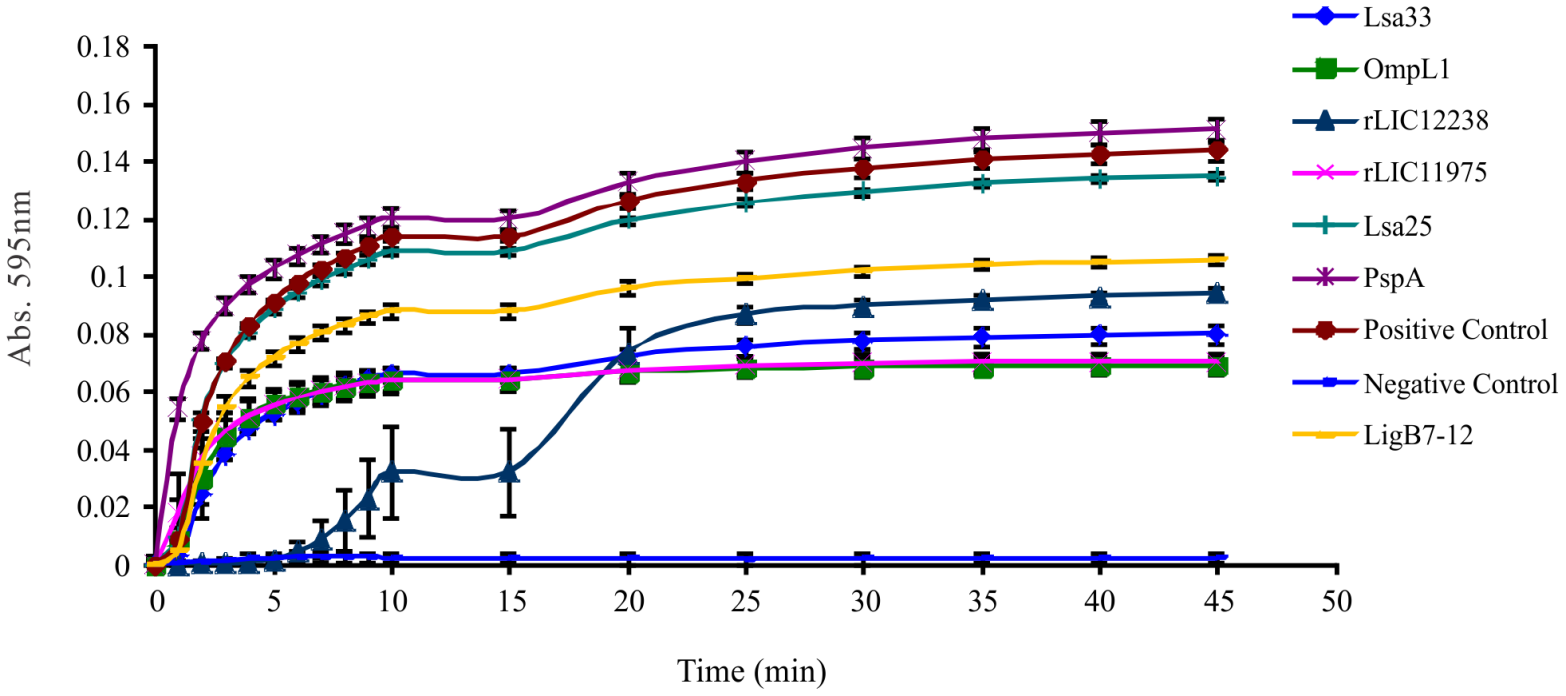
Inhibition of fibrin clot formation by recombinant proteins. Recombinant proteins were resuspended in 0.5 mL of PBS plus 1 mg/mL of Fg and incubated for 2 h at 37°C. The employed concentration of recombinant protein was the one in which there was a binding saturation to Fg: Lsa33 (3,000 nM), OmpL1 (3,000 nM), rLIC12238 (3,500 nM) and rLIC11975 (2,000 nM). The positive control protein LigB7-12 was employed at 1,500 nM, while the negative control proteins, Lsa25 and PspA were used at 3,000 nM. ELISA plates were coated with 90 µL/well of recombinant proteins plus Fg and 10 µL/well of thrombin (10 U/mL). The positive control of the reaction employed Fg (1 mg/mL) plus thrombin (10 U/mL) while in the negative control thrombin was omitted. The fibrin clot formation was measured every 1 min for 10 min and then every 5 min for 35 min. Each point was performed in triplicate and expressed as the mean absorbance value at 595_nm_± SD for each point. Bars represent the mean absorbance values ± standard deviation of three replicates for each condition and are representative of two independent experiments.

Since *Leptospira* bound to Fg inhibited thrombin-catalyzed fibrin formation, we decided to investigate whether the recombinant proteins bound to Fg were capable to mediate this interaction. The concentration of recombinant proteins used was the one in which there was a binding saturation to Fg (Lsa33 – 3,000 nM, OmpL1 – 3,000 nM, rLIC12238 – 3,500 nM and rLIC11975 – 2,000 nM). LigB7-12, previously shown to inhibit thrombin-catalyzed fibrin formation [16], [17], was employed as positive control at 1,500 nM. Recombinant proteins Lsa25 and pneumococcal PspA that do not interact with Fg, were employed as negative controls, at 3000 nM. Each recombinant protein was pre-incubated with 1 mg/mL of Fg at 37°C for 2 h. The reaction mixtures were used to coat ELISA plates and 10 U/mL of thrombin was added per well. The fibrin clot formation was measured every 1 min for 10 min and then every 5 min for 35 min. The percentage of inhibition was calculated as a function of time, taking the complete reaction at the last time-point, in the absence of proteins, as a 100% fibrin formation (positive control). In the negative control, thrombin was omitted from the reaction. The measurements were performed in triplicate and representative curves of two independent experiments are shown in Fig. 6. The proteins rLIC11360 and Lsa30 were not employed on these assays because they were kept at pH 12 for solubility and thrombin is not active above pH 10. The data show that the four proteins tested, Lsa33, rLIC12238, rLIC11975 and OmpL1, elicited an inhibition of 40–50% on the fibrin clot formation, similar to the one elicited by LigB7-12 (Fig. 6). This effect on clot formation by the recombinant proteins was similar with all of them, although a lag time was observed within the rLIC12238 inhibition curve. The inhibition promoted by all proteins was statistically relevant when compared to the positive reaction control. The results are consistent with the K*_D_* of the proteins towards Fg that are in the same order of magnitude. In contrast, Lsa25 and PspA, proteins that do not react with Fg, were not capable to inhibit thrombin-catalyzed fibrin production, resulting in curves similar to the positive control of the reaction (Fig. 6).

### Inhibition of live leptospires binding to Fg by recombinant proteins

**Figure 7:**
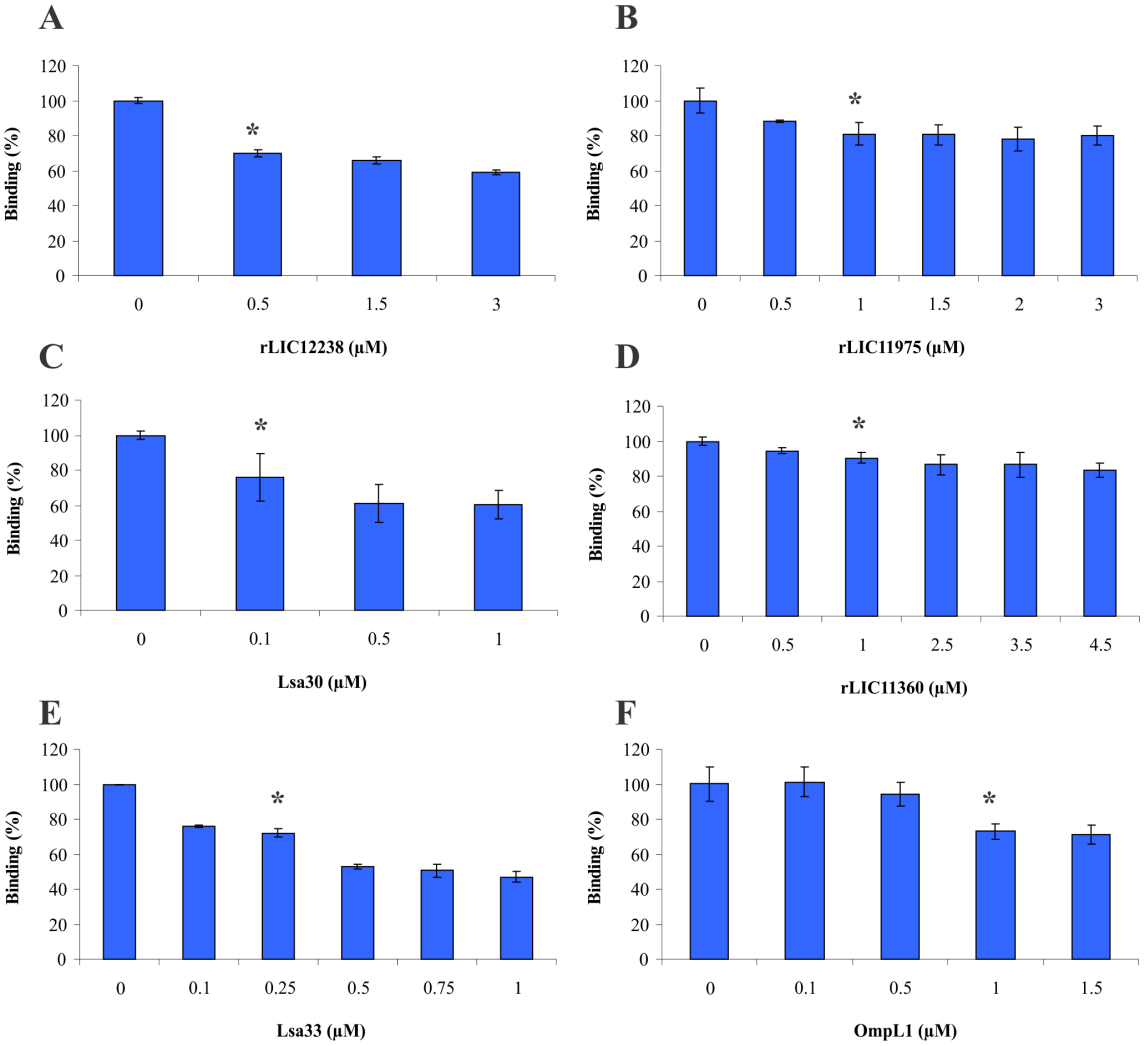
Inhibition of *L. interrogans* attachment to human Fg by recombinant proteins. Fg-coated microtiter wells were incubated with increasing concentration (0–4.5 µM) of rLIC12238 (A), rLIC11975 (B), Lsa30 (C), rLIC11360 (D), Lsa33 (E) and OmpL1 (F) for 90 min prior to the addition of 4×10^7^ leptospires (*L. interrogans* serovar Copenhageni strain M20). Wells were probed with anti-LipL32 serum (1∶4,000). Data are expressed as OD492 _nm_ ± SD of three independent experiments, each performed in triplicate. Significance was assessed by comparison with the “no protein” wells by Students two-tailed *t* test (*P<0.05).

The inhibitory effect exerted by recombinant proteins on leptospiral adherence to Fg was quantified by ELISA. Fg-coated microtiter wells were incubated with increasing concentration (0–4.5 µM) of rLIC12238 (Fig. 7A), rLIC11975 (Fig. 7B), Lsa30 (Fig. 7C), rLIC11360 (Fig. 7D), Lsa33 (Fig. 7E) and OmpL1 (Fig. 7F) for 90 min prior to the addition of 4×10^7^ *L. interrogans* serovar Copenhageni strain M-20. Wells were probed with anti-LipL32 serum, given the fact that LipL32 is a major membrane leptospiral protein [34]. The results are depicted in Figure 7 A to F, and show that the proteins caused a modest, but significant reduction in the number of leptospires adhering to Fg (*P<0.05) with 0.5, 1.0, 1.0, 0.1, 0.25 and 1.0 µM of rLIC12238, rLIC11975, rLIC11360, Lsa30, Lsa33 and OmpL1, respectively. We have performed three independent experiments with comparable results.

## Discussion

Fg is the major clotting protein present in blood plasma with an important role in coagulation and thrombosis. Several studies have suggested roles of Fg and PLG in host bacterial interactions [11]. The fibrinolytic system that generates PLA from PLG and its activators, decomposes the fibrin clot by degrading fibrin [37]. This system is negatively regulated by PLG activator inhibitors. A balance between coagulation system and anticoagulation is necessary to avoid pathophysiological conditions, such as, bleeding and thrombosis [38].

Several Fg-binding proteins of important pathogens, such as, *Staphylococcus aureus* [39], group B *Streptococcus* [40], *Lactobacillus salivarius* [41] and the spirochetes *Treponema pallidum* [42], *T. denticola* [43], [44], [45] have been reported and characterized. These Fg-binding proteins are bacterial surface or secreted adhesins that although acting through different mechanisms will ultimately lead to enhance the bacterial survival in the host [46].

The adhesion of virulent *L. interrogans* strain Fiocruz L1-130 to Fg has been shown to be induced by physiological osmolarity, but the effect of this binding on fibrin clot formation was not evaluated [14]. The leptospiral proteins, LigA and LigB [16], [17] and OmpL37 [15] have been described as Fg-binding proteins. In addition, the interaction of LigB with Fg was shown to inhibit thrombin-catalyzed fibrin formation [16], [17].

In this work, we show that *L. interrogans serovar* Copenhageni are capable of binding human Fg on their surface, as visualized by indirect IFA. Moreover, we have employed ELISA to assess the interaction of human Fg with virulent *L. interrogans* serovar Copenhageni Fiocruz L1-130, attenuated *L. interrogans* serovar Copenhageni M-20 and non-pathogenic saprophytic *L. biflexa* serovar Patoc Patoc 1, and the effect of this binding on the thrombin-catalyzed fibrin formation. Moreover, we studied seven leptospiral adhesin- and PLG-binding proteins, rLIC12238 [18], Lsa33, Lsa25 [19], Lsa30 [20], OmpL1 [21], rLIC11360 and rLIC11975 [22] for their capacity to bind Fg and to inhibit fibrin clot formation. We show that all strains tested bind Fg, including the saprophytic one, and that this interaction inhibits fibrin clot formation. Though the virulent strain appears to be more efficient, the values obtained were not statistically significant. Attachment of recombinant proteins to Fg was specific, dose-dependent and saturable with all proteins but Lsa30. The interaction of the proteins with Fg, similar to the *Leptospira*, promoted an inhibition on thrombin-induced fibrin clot formation. Thus, *Leptospira*, similar to other pathogens, express multiple Fg-binding proteins [38], [46].

As expected, recombinant proteins partially inhibited attachment of intact *L. interrogans* to immobilized Fg. The inhibitory effect exerted by the recombinant proteins was moderate, ranging from 0.1 to 1.0 µM of protein concentration to reach significance, and could be explained by the existence of additional *L. interrogans* binding proteins contributing to the leptospiral adherence to Fg. A correlation between inhibition and affinity was detected with the recombinant protein Lsa33, while no correspondence was seen with the others.

The inhibitory effect on fibrin formation observed with leptospires and with the six recombinant proteins was partial, similar to the LigB, fragment 7–12, employed in this work as positive control, and previously reported by Choy et al., 2011 [16], using LigB fragment 9–11. Our data differ with the total clotting inhibition promoted by the Fg-binding proteins, SdrG of *S. epidermidis* [47], ClfA of *S. aureus* [48] and LigBCen2R [17]. The data suggest that in *Leptospira* other mechanisms might be involved in fibrin formation and/or the main function of these Fg- binding proteins is not associated with the clotting.

We have described the interaction of *Leptospira* with fibrinolytic system and shown that it occurs with virulent, attenuated and saprophyte strains of *Leptospira*. Moreover, we demonstrated that this association renders the bacteria with proteolytic activity capable of degrading ECM components [12]. Several membrane proteins were identified as PLG-binding receptors capable of generating PLA in the presence of activator, suggesting that the interaction with the fibrinolytic system might be important during leptospirosis [49], [18], [50], [36], [19], [21], [20]. Indeed, the increased plasma levels of Fg degradation products detected in leptospirosis has provided evidence for fibrinolysis activity [51], [52]. We show now that *Leptospira* surface-associated PLA activity is capable to degrade Fg *in vitro*, suggesting one possible pathway to generate Fg metabolites during the disease.

Fg is considerably upregulated during inflammation or under exposure to stress such systemic infections [46]. The activation of coagulation cascade with increased levels of plasma Fg during leptospirosis has been detected [53], [51], [54]. It has been suggested that these findings are possibly associated to severe tissue damage, vascular endothelial injury or a compensating production by the liver in response to the augmented Fg utilization [55], [53], [51]. Our data show that *Leptospira* either through their Fg-binding proteins or coated with PLA activity would increase the consumption of Fg molecules by sequestering or degrading them. Under these circumstances, a reduction on fibrin clot formation is expected. In addition, leptospirosis patients with clinical bleeding were reported to have lower platelet counts when compared to other patients [54], a condition that would help decrease thrombosis, facilitate bleeding and help bacterial dissemination.

In conclusion, we have provided molecular evidence of the mechanisms that *Leptospira* could employ to interact with components of the coagulation cascade and the fibrinolytic system. In addition, we have shown that six adhesins could mediate the binding of *Leptospira* to Fg and impair thrombin- induced fibrin clot formation. We believe that our results should contribute to the understanding of the complex coagulopathy observed during leptospirosis.
